# Predictors of quality of life of TB/HIV co-infected patients in the Northern region of Ghana

**DOI:** 10.1186/s12879-024-09247-7

**Published:** 2024-04-12

**Authors:** Jacob Nignan Nabei, Harriet Affran Bonful, Edwin Andrews Afari, Abdul Gafaru Mohammed, Adote Anum

**Affiliations:** 1 Department of Epidemiology and Disease Control, School of Public Health, University of Ghana, Legon, Ghana; 2 Department of Psychology, University of Ghana, Legon, Ghana

**Keywords:** Quality of life, TB/HIV co-infected patients, WHOQOL-HIV BREF, Ghana

## Abstract

**Background:**

Tuberculosis (TB) and human immunodeficiency virus/acquired immunodeficiency syndrome (HIV/AIDS) co-morbidity continues to be a serious worldwide health issue, particularly in Sub-Saharan Africa. Studies on the quality of life (QOL) of TB/HIV co-infected patients guide stakeholders on the delivery of patient-centred healthcare. This study evaluated QOL of TB/HIV co-infected individuals and its contributing factors.

**Methods:**

We conducted a cross-sectional study among TB/HIV co-infected patients, receiving treatment at clinics in the Northern Region of Ghana. Simple random sampling technique was used to select 213 patients from 32 clinics. We gathered information on patients’ QOL using the World Health Organization QOL-HIV BREF assessment tool. At a 5% level of significance, multiple logistic regression analyses were carried out to find correlates of QOL among the patients.

**Results:**

The mean age of the patients was (38.99 ± 14.00) years with most, 33.3% (71/213) aged 30–39 years. Males constituted 54.9% (117/213). About 30.0% (64/213) of the patients reported a good QOL. Being employed (aOR = 5.23, 95% CI: 1.87 – 14.60), and adhering to treatment (aOR = 6.36, 95% CI: 1.51 – 26.65) were significantly associated with a good QOL. Being depressed (aOR = 0.02, 95% CI: 0.03 – 0.29), stigmatized (aOR = 0.31, 95% CI : 0.11 – 0.84), and not exercising (aOR = 0.28, 95% CI: 0.12 – 0.67) were negatively associated with a good QOL.

**Conclusion:**

Less than one-third of TB/HIV co-infected patients in the region have good QOL. To guarantee good QOL, modifiable predictors such as patients’ physical activity and medication adherence should be targeted by the National AIDS and TB Control Programs.

**Supplementary Information:**

The online version contains supplementary material available at 10.1186/s12879-024-09247-7.

## Background

 Tuberculosis (TB) and human immunodeficiency virus (HIV)/acquired immunodeficiency syndrome (AIDS) are diseases of global public health concern, particularly in Sub-Saharan Africa [1]. Despite efforts made to contain TB/HIV co-infection, it continues to impact negatively on the quality of life (QOL) of the infected individuals in developing countries [2]. QOL is defined as individuals’ perception of their position in life in the context of the culture and value systems, with respect to their goals, expectations, standards, and concerns [3]. TB/HIV act in synergy, to affect psychological health, social relationships, spiritual life and other aspects of the life of infected individuals [4].

The COVID-19 pandemic has adversely impacted efforts to deliver crucial services to TB/HIV co-infected patients in order to enhance their quality of life (QOL) [5]. Due to low QOL, this has caused an increase in TB/HIV co-infected person fatalities from 209,000 in 2019 to 214,000 in 2020 globally [6]. The prevalence of TB/HIV co-infection is roughly 21%, and there are now 346,120 people living with HIV/AIDS in Ghana [7]. These people are at a significant risk of dying from a poor quality of life. Studies conducted in Ethiopia and India showed that all (100%) TB/HIV co-infected had poor QOL compared to only HIV/AIDS-infected patients [4, 8, 9]. Unlike the current study, earlier QOL research in Ghana [10, 11], were primarily descriptive in nature and neglected TB/HIV co-infected patients, who had a considerably higher chance of dying from poor QOL.

The QOL of TB/HIV co-infected patients is influenced by a variety of factors such as advanced age, educational level, income level, HIV stage, perceived stigma, depression among others [12, 13]. Psychological distress, a shortened life expectancy, a decreased desire for sexual activity, job loss, grief, poverty, and substance abuse are reported effects of poor QOL among TB/HIV co-infected patients [14, 15].

The Northern Region of Ghana has for the past years recorded a significant number of TB/HIV co-infected patients with a rate of 1.96 patients per 10,000 people (Health Facilities records, Northern Region, 2022). The region has the highest prevalence (80.8%) of multi-dimensional poverty, with poverty index of 0.491 [16]. Majority of people in the area experience poverty and have low quality of life [17, 18].

Thus, this study identified the factors that predicted patients QOL in Ghana’s Northern Region with TB/HIV co-infections. The study’s conclusions will help policymakers prioritize and design focused measures to raise the QOL for TB/HIV co-infected individuals in the area and elsewhere.

## Methods

### Study design

We conducted an analytic cross-sectional study among TB/HIV co-infected patients. Interviews were done using a standardized questionnaire and WHOQOL-BREF instrument to gather patients’ socio-demographic information and to evaluate their QOL respectively.

### Study setting

The study was carried out in the Northern Region of Ghana with 2,310,939 people [19]. The capital city of the area is Tamale, and there are 16 administrative districts. The region is bordered to the north by the North East Region, to the east by the Eastern Ghana-Togo International Border, to the south by the Oti Region, and to the west by the Savannah Region. More than 30 hospitals in the area identify and treat more than 200 TB patients who also co-infected with HIV.

### Study population and eligibility

All patients/participants who were 18 years or older and had received a TB/HIV co-infection diagnosis at a clinic at least one month prior to enrolment, and were willing to participate in the study were enrolled. The study excluded patients who were admitted to wards.

### Sample size determination and sampling process

The sample size was calculated based on the QOL of TB patients in Nigeria [20]. The region recorded 452 TB/HIV co-infected patients. Using Yamane formula for sample size calculation at 95% confidence level and *P* = 0.5 [21];  $\mathrm n=\;\frac N{1+N\left(e\right)^2}$, Where; n = sample size; N = population size = 452 e = precision level = 0.05 Therefore; $n=\;\frac{452}{1+452\left(0.05\right)^2}\;=\;213$. Therefore, the minimum sample size was 213.

### Sampling procedure

All 16 districts in the Northern Region with 32 hospitals that provide services to TB/HIV co-infected patients were selected in the study using total enumerative sampling method. The number of patients sampled was proportionately determined based on size.

At each health facility, simple random sampling method was used to select patients based on proportionate allocation (Table 1).

**Table 1:** Number of TB/HIV patients selected from each facility

Metropolitan/Municipal/District assembly	No. of TB/HIV co-infected patients	Randomly selected patients based on proportionate allocation
Tamale Metropolitan	138	65
Sagnerigu Municipal	63	30
Savelugu Municipal	30	14
Kumbugu District	14	7
Yendi Municipal	49	23
Tolon District	12	6
Nanumba North Municipal	32	15
Nanumba South District	23	11
Tatale-Sanguli District	18	8
Zabzugu District	12	6
Gushiegu Municipal	14	7
Kpandai District	15	7
Nanton District	13	6
Karaga District	12	5
Saboba District	3	1
Mion District	4	2
** Total**	** 452**	** 213**

List of all the patients at each facility was obtained from patients’ registers. All the selected patients at each facility were contacted by healthcare workers through their telephone numbers. Patients were told to report to facilities on a particular day and interviewed by research assistants at a private place after consenting.

### Study instruments

WHOQOL-BREF assessment tool was used to assess the QOL of study participants. It is an abbreviated form of the WHOQOL-100 assessment tool, made up of 26 items on QOL in physical health, psychological well-being, social relationships and environmental health domains. See Supplementary 1, Additional File 1.

Kessler Psychological Distress Scale (K10) was used to measure depression of study participants. The scale is made up of 10 questions about the emotional states of participants each with a 5-level response scale from 1 ‘none of the time’ to 5 ‘all of the time’. Questions 3 and 6 are not asked provided the response to the preceding question was ‘none of the time’, in which case they will be automatically scored 1. Scores of all 10 items are added giving a minimum score of 10 and a maximum score of 50 [22]. See Supplementary 2, Additional File 2.

Perceived stigma was measured with a 6-item Internalized AIDS-Related Stigma Scale (IA-RSS) as described in Supplementary 3, Additional File 3. Scale scores were summed to obtain composite stigma scores and those who scored below the mean were labelled as ‘not stigmatized’ and those above the mean as ‘stigmatized’ [23].

Adherence to medications was assessed using the 5-item AIDS Clinical Trials Group (ACTG) Adherence Self Report Tool. The questionnaire queries the patient about the number of doses missed of medication during each of the 4 days before a clinic visit. Because patients usually take more than 1 drug, the adherence percentage for each of the 4 days before visiting the clinic was calculated as [1-(number of missed dosage units/number of dosage units prescribed] ×100. The adherence percentage was also dichotomized into “adherent” (≥ 95%) or “non-adherent” (˂ 95%) [24, 25]. See Supplementary 4, Additional File 4.

### Validity and reliability of study instruments

The WHOQOL-BREF, K10, IA-RSS and the ACTG instruments used in the study are valid, widely used and internationally accepted. Also, pre-testing with respondents using the instruments was performed. The WHOQOL-BREF reliability was determined using the Cronbach’s alpha (α). Internal consistency of items in the various domains was considered when the α ≥ 0.7 [26].

### Data collection

Three (3) field assistants with a minimum of higher national diploma (HND) degree were recruited and trained by the principal investigator (PI) on data collection tools and process for 4 days. Debriefing meetings were held with the interviewers and research team to identify and address any problems identified during the pretesting. The research team led by the PI conducted the pretest of data collection tools. Private spaces at various facilities were used to collect respondents’ relevant information using all the data collection tools. The interviews lasted 30 minutes on average and the data were collected over a period of 4 months. 

### Data management and analysis

Data collected using KoBoCollect questionnaire were extracted into Microsoft Excel, cleaned and validated. Data were analysed using STATA version 15.1. Descriptive statistics were performed by estimating frequencies and percentages for categorical data. Means and Standard deviations were computed for normally distributed continuous data.

In assessing QOL using WHOQOL-BREF assessment tool, domain scores were calculated using Excel. Mean scores were obtained by adding all the items within each domain and dividing by number of items in that domain. Domain score was obtained by multiplying the mean score by 4 [27]. To ensure that the domain scores are comparable with the scores used in the WHOQOL-100, the scores were transformed to a 0-100 scale by employing the formula:

$\mathrm{Transformed}\;\mathrm{score}\;=\;(\mathrm{score}-4)\;\mathrm x\;(100/16)$ [27]. QOL was dichotomized as 'poor' and 'good'. This was done by using the mean for each domain as a cut-off point. Individuals with scores greater than the mean were considered as having  good QOL whilst individuals with a score less than the mean were considered as having  poor QOL.

Multiple logistic regression was used to determine predictors of QOL at a 5% significant level.

## Results

### Background characteristics of TB/HIV co-infected patients, Northern region

A total of 213 respondents were included in the study. The mean age of the respondents was (38.99 ± 14.00) years with most, 33.3% (71) aged 30-39 years. More than half, 54.9% (117) of the respondents were males. More over two-thirds of respondents, or 72.8% (155), were married, and 61.0% (130) identified as Muslims. Less than a quarter, or 21.6% (46) of the respondents, were working (Table 2).

**Table 2:** Socio-demographic characteristics of TB/HIV co-infected patients, Northern region

Variables	Frequency (n) *N* = 213	Percentages (%)
**Age (Mean ± SD)**	38.99 ± 14.00	
**Age (years)**		
< 20	9	4.2
20–29	44	20.7
30–39	71	33.3
40–49	51	23.9
50–59	15	7.0
60–69	15	7.0
≥ 70	8	3.8
**Sex**		
Female	96	45.1
Male	117	54.9
**Marital status**		
Married	155	72.8
Single	58	27.2
**Religion**		
Christian	83	39.0
Islam	130	61.0
**Educational level**		
No formal education	37	17.4
Primary school	39	18.3
JHS/Middle school	15	7.0
SHS/A level/Tech/Voc	78	36.6
Tertiary	44	20.7
**Current employment status**		
Not employed	167	78.4
Employed	46	21.6
**Employment type**		
Government	20	43.5
Private self	16	34.8
Private non-self	10	21.7
**Residency**		
Rural	23	10.8
Peri-urban	45	21.1
Urban	145	68.1

### Behavioral factors and other lifestyle history of TB/HIV co-infected patients, Northern region 

Seventy-six percent 76% (161) of the 213 respondents had depression. Nearly half of those, 47.8% (77) had mild depression, and 18.6% (30) had severe depression. The majority of respondents, or 81.7% (174), stated that they have experienced stigma because of their medical issues. Nearly two-thirds of respondents, or 67.3% (142), reportedly exercised, while more than three-fourths, or 78.9% (168), indicated they took their medications as prescribed. (Table 3).

**Table 3:** Behavioral factors and other lifestyle histories of TB/HIV co-infected patients, Northern region

Variables	Frequency (n) *N* = 213	Percentages (%)
**Depression status**		
Not depressed	52	24.4
Mildly depressed	77	36.2
Moderately depressed	54	25.3
Severely depressed	30	14.1
**Stigma status**		
Not stigmatized	39	18.3
Stigmatized	174	81.7
**Smoking status**		
Not smoking	188	89.1
Smoking	23	10.9
**Alcohol intake**		
Non-alcoholic	180	85.3
Alcoholic	31	14.7
**Exercise status**		
Not exercising	69	32.7
Exercising	142	67.3
**Adherence to medications**		
Non-adherence	45	21.1
Adheres to medications	168	78.9

### Distribution of WHOQOL-BREF domains scores of TB/HIV co-infected patients, Northern region 

The mean scores and standard deviations for QOL domains from highest to lowest among respondents were environmental health (59.40 ± 16.70), physical health (55.70 ± 13.64), psychological health (54.89 ± 10.86), and social relationships (47.46 ± 17.17) (Table 4).

**Table 4:** Distribution of WHOQOL-BREF domains scores of TB/HIV co-infected patients, Northern region

Quality of life domains	Mean (± SD) domain score	Cronbach’s alpha
Physical health	55.70(13.64)	0.75
Psychological health	54.89(10.86)	0.78
Social relationships	47.46(17.17)	0.65
Environmental health	59.40(16.70)	0.70

### Distribution of self-rated quality of life (QOL) according to WHOQOL-BREF domains for TB/HIV co-infected patients, Northern region 

According to the WHOQOL-BREF domains, the bulk of respondents who self-rated their QOL to be poor, 64.3% (137) were found in the environmental health domain. Social relationship domain however, recorded highest, 53.5% (114) number of respondents with good self-rated QOL. Generally, most respondents had poor self-rated QOL in all domains except in social domain (Table 5).

**Table 5:** Distribution of self-rated QOL according to WHOQOL-BREF domains for TB/HIV co-infected patients, Northern region

Quality of life domains	Poor n(%)	Good n(%)
Physical health	117(54.9)	96(45.1)
Psychological health	121(56.8)	92(43.2)
Social relationships	99(46.5)	114(53.5)
Environmental health	137(64.3)	76(35.7)

### Distribution of WHOQOL-BREF domains quality of life (QOL) and overall QOL of TB/HIV co-infected patients, Northern region

Of the 213 respondents studied, 70.0% (149) had their overall QOL to be poor across all domains of the WHOQOL-BREF assessment tool. Of the 117 respondents who self-rated their QOL to be poor with respect to physical health domain, almost all, 88.0% (103) of them also had their overall QOL to be poor (Table 6).

**Table 6:** Distribution of WHOQOL-BREF domains QOL and overall QOL of TB/HIV co-infected patients, Northern region

QOL domains	Overall QOL	
	**Poor** **n(%)**	**Good** **n(%)**
**Physical health**		
Poor QOL (Self-rated)	103(88.0)	14(12.0)
Good QOL (Self-rated)	46(47.9)	50(52.1)
**Psychological health**		
Poor QOL (Self-rated)	114(94.2)	7(5.8)
Good QOL (Self-rated)	35(38.0)	57(62.0)
**Social relationships**		
Poor QOL (Self-rated)	91(91.9)	8(8.1)
Good QOL (Self-rated)	58(50.9)	56(49.1)
**Environmental health**		
Poor QOL (Self-rated)	119(86.9)	18(13.1)
Good QOL (Self-rated)	30(39.5)	46(60.5)

### Factors associated with overall QOL of TB/HIV co-infected patients, Northern region

At the crude stage of the multivariate logistic regression analysis, variables that were suggestive to be significantly associated with the overall QOL were religion, level of education, sex, marital status, current employment status, the main mode of transport to and from clinic, depression, stigma, exercise, adherence to medications and out-pocket-purchase of drugs. Respondents who were single had 5.2 times increased odds of good QOL compared to those who were married (cOR = 5.19, 95% CI: 2.11 – 12.82, *p* < 0.000). Respondents who had tertiary education had 5.7 times increased odds of good QOL as against those who did not have any formal education (cOR = 5.71, 95% CI: 1.72 – 18.95, *p* < 0.004). Similarly, compared with those who were unemployed, employed respondents had 5.7 times increased odds of good QOL (cOR = 5.66, 95% CI: 2.82 – 11.37, *p* < 0.000). Respondents who had mild depressive conditions had 71% reduced odds of having good QOL in comparison to those who were not depressed (cOR = 0.29, 95% CI: 0.14 – 0.62, *p* < 0.001). Similarly, compared to those who were not depressed, respondents who had severe depressive conditions experienced a reduction of 97.5% less in the odds of having good QOL (cOR = 0.03, 95% CI: 0.03 – 0.20, *p* < 0.000). Also, respondents who reportedly adhere to their medications had about 8 times increased odds of good QOL compared to their counterparts (cOR = 7.98, 95% CI: 2.37 – 26.84, *p* < 0.001).

After adjusting for other variables, religion, education level, sex, marital status, current employment status, primary mode of transportation to and from the clinic, depression, stigma, exercise, and adherence to medications remained associated with overall QOL. Respondents who had tertiary education had 6.3 times increased odds of good QOL compared to their counterparts (aOR = 6.32, 95% CI: 1.64 – 24.38, *p* < 0.007). Respondents who were gainfully employed had 5.2 times increased odds of good QOL compared to their unemployed counterparts (aOR = 5.23, 95% CI: 1.87 – 14.60, *p* < 0.002). Also, compared to those who were not depressed, mildly depressed patients had a reduction of 80% in the odds of having good QOL) (aOR = 0.20, 95% CI: 0.08 – 0.52, *p* < 0.001), whilst those who were severely depressed experienced a reduction of 97% in the odds of having good QOL (aOR = 0.03, 95% CI:0.03 – 0.29, *p* < 0.003). Similarly, respondents who had perceived stigma had a reduction of 69% in the odds of having good QOL compared to those who were stigmatized (aOR = 0.31, 95% CI: 0.11 – 0.85, *p* < 0.022). Furthermore, respondents who reportedly adhere to their medications had 6.4 times increased odds of good QOL compared to their counterparts (aOR = 6.36, 95% CI: 1.52 – 26.65, *p* < 0.011)(Table 7).

**Table 7:** Factors associated with overall QOL of TB/HIV co-infected patients, Northern region

Variables	Overall QOL		**cOR(95% CI)**	***p*-value**	**aOR(95% CI)**	***p*-value**
	Poor *n* = 149(%)	Good *n* = 64(%)				
**Sex**						
Female	59(61.5)	37(38.5)	Ref		Ref	
Male	90(76.9)	27(23.1)	0.48(0.26–0.87)	0.015*	0.42(0.20–0.88)	0.021**
**Marital status**						
Married	97(62.6)	58(37.4)	Ref		Ref	
Single	52(89.7)	6(10.3)	5.18(2.10–12.81)	0.000*	9.69(3.08–30.51)	0.001**
**Religion**						
Christian	44(53.0)	39(47.0)	Ref		Ref	
Islam	105(80.8)	25(19.2)	0.27(0.15–0.50)	0.000*	0.19(0.09–0.42)	0.001**
**Educational level**						
No formal education	33(89.2)	4(10.8)	Ref		Ref	
Primary school	28(71.8)	11(28.2)	3.24(0.93–11.32)	0.065	6.95(1.65–29.23)	0.008**
JHS/Middle school	6(40.0)	9(60.0)	12.38(2.86–53.51)	0.001*	41.63(7.54–229.89)	0.001**
SHS/A-level/Tech/Voc	56(71.8)	22(28.2)	3.24(1.03–10.23)	0.045*	6.16(1.70–22.34)	0.006**
Tertiary	26(59.1)	18(40.9)	5.71(1.72–18.95)	0.004*	6.32(1.64–24.38)	0.007**
**Current employment status**						
Not employed	131(78.4)	36(21.6)	Ref		Ref	
Employed	18(39.1)	28(60.9)	5.66(2.82–11.37)	0.000*	5.23(1.87–14.60)	0.002**
**Employment type**						
Government	4(20.0)	16(80.0)	Ref		Ref	
Private self	6(37.5)	10(62.5)	0.06(0.01–0.42)	0.004*	0.07(0.01–0.61)	0.016
Private non-self	8(80.0)	2(20.0)	0.42(0.09–1.85)	0.250	0.78(0.14–4.26)	0.775
**Average monthly income (GH¢)**						
≤ 500	98(72.6)	37(27.4)	Ref		Ref	
501–1000	29(80.6)	7(19.4)	0.64(0.26–1.59)	0.334	1.19(0.64–2.20)	0.586
1001–1500	9(100.0)	0(0.0)	-	-	-	-
1501–2000	8(100.0)	0(0.0)	-	-	-	-
> 2000	5(20.0)	20(80.0)	10.60(3.71–30.29)	0.000	-	-
**Residency**						
Rural	17(73.9)	6(26.1)	Ref		Ref	
Peri-urban	39(86.7)	6(13.3)	0.44(0.12–1.55)	0.199	0.55(0.14–2.14)	0.388
Urban	93(64.1)	52(35.9)	1.58(0.59–4.27)	0.363	1.54(0.50–4.78)	0.451
**Transport**						
Private vehicle	1(11.1)	8(88.9)	Ref		Ref	
Commercial motorcycle	89(70.1)	38(29.9)	0.05(0.01–0.44)	0.007*	0.06(0.01–0.48)	0.009**
Commercial bus	59(79.7)	15(20.3)	0.03(0.01–0.27)	0.002*	0.04(0.01–0.35)	0.004**
Other means	0(0.00)	3(100.0)	-	-	-	-
**Depression status**						
Not depressed	22(42.3)	30(57.7)	Ref		Ref	
Mildly depressed	55(71.4)	22(28.6)	0.29(0.14–0.62)	0.001*	0.20(0.08–0.52)	0.001**
Moderately depressed	43(79.6)	11(20.4)	0.19(0.08–0.44)	0.000*	0.15(0.05–0.45)	0.001**
Severely depressed	29(96.7)	1(3.3)	0.03(0.01–0.20)	0.000*	0.029(0.01–0.29)	0.003**
**Stigma status**						
Not stigmatized	17(43.6)	22(56.4)	Ref		Ref	
Stigmatized	132(75.9)	42(24.1)	0.25(0.12–0.51)	0.000*	0.31(0.11–0.85)	0.022**
**Exercise status**						
Exercises	109(76.8)	33(23.2)	Ref		Ref	
Do not exercise	38(55.1)	31(44.9)	0.37(0.20–0.69)	0.002*	0.29(0.12–0.67)	0.004**
**Adherence to medications**						
Non-adherence	42(93.3)	3(6.7)	Ref		Ref	
Adherence	107(63.7)	61(36.3)	7.98(2.37–26.84)	0.001*	6.36(1.52–26.65)	0.011**
**Out of pocket purchase of drugs**						
No	145(71.4)	58(28.6)	Ref		Ref	
Yes	2(25.0)	6(75.0)	7.50(1.47–38.24)	0.015*	7.70(1.50–39.52)	0.015**
**TB category**						
Category 1	147(71.0)	60(29.0)	Ref		Ref	
Category 2	2(33.3)	4(66.7)	4.90(0.87–27.47)	0.071	6.76(0.96–47.74)	0.055

*cOR* Crude odds ratio, *aOR* Adjusted odds ratio, *CI* Confidence Interval, *QOL* Quality of life

*Statistically significant before adjustment

**Statistically significant after adjustment

## Discussion

The study examined the quality of life (QOL) of TB/HIV co-infected patients in the Northern Region of Ghana. The WHOQOL-BREF assessment tool used to determine the QOL of the patients had good internal consistency because all the domains had Cronbach’s alpha equal to or more than 0.7 (α ≥ 0.7) [26].

The majority of respondents reported poor QOL, which could be attributed to the study participants’ subpar socioeconomic circumstances. Many people in Ghana’s Northern Region live in deplorable conditions, with significant rates of unemployment and illiteracy, which may have a severe effect on the patients’ QOL. This result is in line with those of related studies in Ethiopia and India [4, 8, 9]. Similar studies conducted in Brazil, however, revealed that patients with TB, HIV and both conditions appear to have similar QOL [28].

The study found that women had better QOL compared to men, probably due to men’s health-seeking behaviors. Men typically present to the hospital at the latter stage of their illnesses, which facilitates poor disease outcomes and poor QOL. Also, in Ghanaian societies particularly, in the Northern Region, men are solely responsible for the provision of food, shelter, payment of children's school fees, utility bills among others [29]. These could lead to the development of other health conditions like hypertension and depression thereby making their QOL poor.   This finding corroborates the results of similar studies in Ghana [11] as well as in Uganda and Ethiopia [30, 31], but disagrees with results from Burkina Faso and Ethiopia [9, 32, 33]. These differences could be due to the different socio-economic situations of respondents in the various areas.

The results of this study showed that, unmarried patients had good overall QOL in all domains. This may be the result of anxiety and tension brought on by married couples in which one partner accuses the other of causing TB, HIV/AIDS, or both. Social responsibilities that married couples bear could impact negatively on their QOL. This finding is supported by other studies conducted in Ethiopia and India [34, 35]. In contrast with the present study, previous studies revealed that a lack of family support as a result of being single leads to poor QOL [8, 9].

One’s QOL might be impacted by their religious practice, particularly in the spiritual area. Oliveira and colleagues (2015) found that religion and personal beliefs, were positively connected with having a good QOL among people living with HIV/AIDS [36]. Individuals from different religious backgrounds have different beliefs and superstitions attached to various diseases, hence the reason for the disparities in the QOL of Muslims and Christians in this study. However, further research is necessitated to unearth the main reasons why Muslims in this study had reduced QOL compared to their Christian counterparts.

Compared to their uneducated peers, literate TB patients with HIV/AIDS would be more informed of their diseases and expected to faithfully follow their drug regimens. This will improve their QOL. Additionally, educated patients are more likely than uneducated people to find long-term employment that will pay well, easily access technologies that can improve their QOL. These are in line with the results of earlier studies carried out in other nations [4, 9, 13, 14]. Contrasting results were rather found by Beke and colleagues, where education was not significantly associated with good QOL in their study [37].

People who are currently employed have better QOL, probably due to their positive psychological health as a result of their income, job stability, and job satisfaction. This enables them to have good nutrition that can boost their immune status and subsequently improve their QOL, consistent with comparable studies conducted in South Africa, Ethiopia and Canada [38–41]. Conversely, Jha et al., (2019) found no association between the source of income and QOL [4]. These differences could be explained by the fact that most of the respondents were unemployed partly due to the unavailability of jobs in the region.

Depression and stigma being predictors of poor QOL was not surprising because TB and HIV are both stigmatizing diseases that act synergistically to have adverse effects on the overall QOL. Interestingly, our findings revealed that the more depressed a patient is, the worse his/her QOL. Lack of support of all forms due to unwillingness of such patients to disclose their status increases the risk of developing depression. Similar findings are reported in studies conducted in Ethiopia [8, 9, 35], Nigeria [3] and Nepal [14], but contrary to what Jha et al., (2019) found in India where there was no association between depression with QOL [4].

Physical exercise predicts good QOL of TB/HIV co-infected patients. This could be explained by the fact that exercise lowers the risk of development of non-communicable diseases like depression and others, thereby improving their general health, and psychological thinking [42]. Results from several studies conducted in both developing and developed countries confirmed this finding [43–46].

Non-adherence to medications predicted poor QOL because this increases the risk of relapse, resistance to drugs, prolonged infectiousness and death subsequently. Possible factors that could have accounted for non-adherence to medications include but are not limited to long distances to treatment centres and no or inadequate counselling on medications. The finding is consistent with the studies conducted in other settings [38, 47–49], but in contrast with what was found in other studies [50, 51].

This study has some possible weaknesses. The WHOQOL-BREF instrument covered only four domains, which may have made it less thorough than utilizing six domains. Also, the study did not assess other co-morbidities like diabetes and hypertension, which may impact on the QOL. Furthermore, being a cross-sectional study involving the use of patient self-report tools, recall bias may have been introduced. This was minimized by giving respondents ample time to refresh their memories before answering a question. Self-report on health conditions has also been shown to give accurate measure [52].

## Conclusion

The majority of TB/HIV patients in Ghana’s Northern Region had low quality of life (QOL) across all WHOQOL-BREF assessment domains. The respondents’ sex, marital status, religion, amount of education, employment status, depression, stigma, physical activity, and medication adherence continue to be QOL predictors. The National AIDS and TB Control Programs as well as other concerned organizations or institutions can consider implementing robust strategies and policies that promotes exercising  systematically  and adherence to medications among TB/HIV co-infected patients.

### Supplementary Information

**Supplementary Material 1.**

**Supplementary Material 2.**

**Supplementary Material 3.**

**Supplementary Material 4.**

## Data Availability

The study data is available upon request from the corresponding author (habonful@ug.edu.gh).

## Abbreviations

AIDS: Acquired Immunodeficiency Syndrome
ARV: Antiretroviral
COVID-19: Coronavirus disease
DOTS: Directly Observed Treatment, Short-Course
HIV: Human Immunodeficiency Virus
HRQOL: Health-Related Quality of Life
IA-RSS: Internalized AIDS- Related Stigma Scale (IA-RSS)
K10: Kessler Psychological Distress Scale
NACP: National AIDS/STI Control Programme
NTP: National TB Control Programme
PLHIV: People Living With HIV/AIDS
QOL: Quality of Life
SF-36: Short-form (36-item) Quality of Life assessment instrument
STI: Sexually Transmitted Infection
TB: Tuberculosis
TB/HIV: TB and HIV
WHO: World Health Organization
WHOQOL: World Health Organization Quality of Life
WHOQOL-BREF: Short version (26-item) World Health Organization Quality of Life-100 assessment instrument
