# Supplementary material for: Predictors of quality of life of TB/HIV co-infected patients in the Northern region of Ghana
Source: BMC Infect Dis. 2024 Apr 12;24:396. doi: 10.1186/s12879-024-09247-7 (PMC11010380; doi:10.1186/s12879-024-09247-7)
Supplement: Supplementary file 1 — Supplementary Material 1. [file 12879_2024_9247_MOESM1_ESM.docx]

**Additional File 1**

**Supplementary 1**

**WHO Quality of life (WHOQOL) –BREF Assessment Tool**

*(Information below would be read to respondent)*

The following questions ask how you feel about your quality of life, health, or other areas of your life. **Please, answer all the questions**. **Please choose the answer** that appears most appropriate. If you are not sure about which response to give to a question, the first response you think of is often the best one.

Please keep in mind your standards, hopes, pleasures and concerns. We ask that you think about your life **in the last four weeks.**

|  |  | Very poor | Poor | Neither poor nor good | Good | Very good |
| --- | --- | --- | --- | --- | --- | --- |
| 1 | How would you rate your quality of life? | 1 | 2 | 3 | 4 | 5 |

|  |  | Very dissatisfied | dissatisfied | Neither satisfied nor dissatisfied | Satisfied | Very satisfied |
| --- | --- | --- | --- | --- | --- | --- |
| 2 | How satisfied are you with your health? | 1 | 2 | 3 | 4 | 5 |

The following questions ask about **how much** you have experienced certain things in the last four weeks.

|  |  | Not at all | A little | A moderate amount | Very much | An extreme amount |
| --- | --- | --- | --- | --- | --- | --- |
| 3 | To what extent do you feel that physical pain prevents you from doing what you need to do? | 5 | 4 | 3 | 2 | 1 |
| 4 | How much do you need any medical treatment to function in your daily life? | 5 | 4 | 3 | 2 | 1 |
| 5 | How much do you enjoy life? | 1 | 2 | 3 | 4 | 5 |
| 6 | To what extent do you feel your life to be meaningful? | 1 | 2 | 3 | 4 | 5 |

|  |  | Not at all | A little | A moderate amount | Very much | Extremely |
| --- | --- | --- | --- | --- | --- | --- |
| 7 | How well are you able to concentrate? | 1 | 2 | 3 | 4 | 5 |
| 8 | How safe do you feel in your daily life? | 1 | 2 | 3 | 4 | 5 |
| 9 | How healthy is your physical environment? | 1 | 2 | 3 | 4 | 5 |

The following questions ask about **how completely** you experience or were able to do certain things in the last four weeks.

|  |  | Not at all | A little | Moderately | Mostly | Completely |
| --- | --- | --- | --- | --- | --- | --- |
| 10 | Do you have enough energy for everyday life? | 1 | 2 | 3 | 4 | 5 |
| 11 | Are you able to accept your bodily appearance? | 1 | 2 | 3 | 4 | 5 |
| 12 | Have you enough money to meet your needs? | 1 | 2 | 3 | 4 | 5 |
| 13 | How available to you is the information that you need in your day-to-day life? | 1 | 2 | 3 | 4 | 5 |
| 14 | To what extent do you have the opportunity for leisure activities? | 1 | 2 | 3 | 4 | 5 |

|  |  | Very poor | Poor | Neither poor nor good | Good | Very good |
| --- | --- | --- | --- | --- | --- | --- |
| 15 | How well are you able to get around? | 1 | 2 | 3 | 4 | 5 |

The following questions ask you to say how good or satisfied you have felt about various aspects of your life over the last four weeks.

|  |  | Very dissatisfied | dissatisfied | Neither satisfied nor dissatisfied | Satisfied | Very satisfied |
| --- | --- | --- | --- | --- | --- | --- |
| 16 | How satisfied are you with your sleep? | 1 | 2 | 3 | 4 | 5 |
| 17 | How satisfied are you with your ability to perform your daily living activities? | 1 | 2 | 3 | 4 | 5 |
| 18 | How satisfied are you with your capacity for work? | 1 | 2 | 3 | 4 | 5 |
| 19 | How satisfied are you with yourself? | 1 | 2 | 3 | 4 | 5 |
| 20 | How satisfied are you with your personal relationships? | 1 | 2 | 3 | 4 | 5 |
| 21 | How satisfied are you with your sex life? | 1 | 2 | 3 | 4 | 5 |
| 22 | How satisfied are you with the support you get from your friends? | 1 | 2 | 3 | 4 | 5 |
| 23 | How satisfied are you with the conditions of your living place? | 1 | 2 | 3 | 4 | 5 |
| 24 | How satisfied are you with your access to health services? | 1 | 2 | 3 | 4 | 5 |
| 25 | How satisfied are you with your transport? | 1 | 2 | 3 | 4 | 5 |

The following question refers to how often you have felt or experienced certain things in the last four weeks.

|  |  | Never | Seldom | Quite often | Very often | Always |
| --- | --- | --- | --- | --- | --- | --- |
| 26 | How often do you have negative feelings such as blue mood, despair, anxiety, depression? | 5 | 4 | 3 | 2 | 1 |

Do you have any comments about the assessment?...........................................................................

………………………………………………………………………………………………………

Question 27 to 30 to be completed at the end of the interview session.

|  |  | Equation for computing domain scores (Raw scores) | Mean scores | Domain scores (Mean score * 4) | Transformed scores to 0-100 scale  (Score-4)*(100/16) |
| --- | --- | --- | --- | --- | --- |
| 27 | Domain 1 | (6-Q3) + (6-Q4) + Q10 + Q15 + Q16 + Q17 + Q18 |  |  |  |
| 28 | Domain 2 | Q5 + Q6 + Q7 + Q11 + Q19 + (6-Q26) |  |  |  |
| 29 | Domain 3 | Q20 + Q21 + Q22 |  |  |  |
| 30 | Domain 4 | Q8 + Q9 + Q12 + Q13 + Q14 + Q23 + Q24 +Q25 |  |  |  |

Thank you for your cooperation.
