# Supplementary material for: Predictors of quality of life of TB/HIV co-infected patients in the Northern region of Ghana
Source: BMC Infect Dis. 2024 Apr 12;24:396. doi: 10.1186/s12879-024-09247-7 (PMC11010380; doi:10.1186/s12879-024-09247-7)
Supplement: Supplementary file 2 — Supplementary Material 2. [file 12879_2024_9247_MOESM2_ESM.docx]

**Additional File 2**

**Supplementary 2**

**Kessler Psychological Distress Scale (K10)**

Please, tick the answer that is correct for you

|  | **All of the time (score 5)** | **Most of the time (score 4)** | **Some of the time (score 3)** | **A little of the time (score 2)** | **None of the time (score 1)** |
| --- | --- | --- | --- | --- | --- |
| 1. In the past 4 weeks, about how often did you feel tired out for no good reason? |  |  |  |  |  |
| 1. In the past 4 weeks, about how often did you feel nervous? |  |  |  |  |  |
| 1. In the past 4 weeks, about how often did you feel so nervous that nothing could calm you down? |  |  |  |  |  |
| 1. In the past 4 weeks, about how often did you feel hopeless? |  |  |  |  |  |
| 1. In the past 4 weeks, about how often did you feel restless or fidgety? |  |  |  |  |  |
| 1. In the past 4 weeks, about how often did you feel so restless you could not sit still? |  |  |  |  |  |
| 1. In the past 4 weeks, about how often did you feel depressed? |  |  |  |  |  |
| 1. In the past 4 weeks, about how often did you feel that everything was an effort? |  |  |  |  |  |
| 1. In the past 4 weeks, about how often did you feel so sad that nothing could cheer you up? |  |  |  |  |  |
| 1. In the past 4 weeks, about how often did you feel worthless? |  |  |  |  |  |

Sum all the 10 items scores and interpret (likelihood of having mental disorder/psychological distress) as follows:

- **10-19 Likely to be well**
- **20-24 Likely to have a mild disorder**
- **25-29 Likely to have moderate disorder**
- **30-50 Likely to have a severe disorder**

Thank you for your cooperation
