# Supplementary material for: Predictors of quality of life of TB/HIV co-infected patients in the Northern region of Ghana
Source: BMC Infect Dis. 2024 Apr 12;24:396. doi: 10.1186/s12879-024-09247-7 (PMC11010380; doi:10.1186/s12879-024-09247-7)
Supplement: Supplementary file 3 — Supplementary Material 3. [file 12879_2024_9247_MOESM3_ESM.docx]

**Additional File 3**

**Supplementary 3**

**Internalized AIDS-Related Sigma Scale**

Please, tick the answer that is correct for you.

|  | **Agree** | **Disagree** |
| --- | --- | --- |
| 1. It is difficult to tell people about my HIV infection |  |  |
| 1. Being HIV positive makes me feel dirty |  |  |
| 1. I feel guilty that I am HIV positive |  |  |
| 1. I am ashamed that I am HIV positive. |  |  |
| 1. I sometimes feels worthless because I am HIV positive. |  |  |
| 1. I hide my HIV status from others |  |  |

Thank you.
