# Supplementary material for: Predictors of quality of life of TB/HIV co-infected patients in the Northern region of Ghana
Source: BMC Infect Dis. 2024 Apr 12;24:396. doi: 10.1186/s12879-024-09247-7 (PMC11010380; doi:10.1186/s12879-024-09247-7)
Supplement: Supplementary file 4 — Supplementary Material 4. [file 12879_2024_9247_MOESM4_ESM.docx]

**Additional File 4**

**Supplementary 4**

**AIDS Clinical Trials Group (ACTG) Adherence Self Report Tool**

1. Are you currently expected to be taking any medications? a) Yes b) No (If No, stop)
2. Have you missed (taking only a portion of a dose) taking any of the prescribed medications over the last four days? a) Yes b) No (If no go to Q.4)
3. If Yes to Q.2 above, please, complete the table below indicating the drug(s), number of prescribed doses (not number of pills) and how long you have missed taking over the last four days

|  |  |  |  |  |  |  |
| --- | --- | --- | --- | --- | --- | --- |
| Name of drug | No. of prescribed doses per day | No. of dose(s) missed | 1 day ago | 2 days ago | 3 days ago | 4 days ago |
|  |  |  |  |  |  |  |
|  |  |  |  |  |  |  |
|  |  |  |  |  |  |  |

1. How closely did you follow your specific medications schedule over the last four days? Tick in appropriate box

Never Some of the time About half of the time Most of the time All of the time

0 1 2 3 4

1. Do any of your medications have special instructions, such as “take with food” or “on an empty stomach” or “with plenty of fluids”? a) Yes b) No (If no go to Q.7)
2. If Yes, how often did you follow those special instructions over the last four days?

Never Some of the time About half of the time Most of the time All of the time

0 1 2 3 4

1. When was the last time you missed any of your medications? Tick the appropriate box

Within the past week 5

1-2 weeks ago 4

3-4 weeks ago 3

About 1-3 months ago 2

More than 3 months ago 1

Never skip medications 0

Thank you very much
